# Progesterone Intramuscularly or Vaginally Administration May Not Change Live Birth Rate or Neonatal Outcomes in Artificial Frozen-Thawed Embryo Transfer Cycles

**DOI:** 10.3389/fendo.2020.539427

**Published:** 2020-12-04

**Authors:** Yuan Liu, Yu Wu

**Affiliations:** Reproductive Medicine Center, Department of Obstetrics and Gynecology, Shanghai General Hospital, Shanghai Jiaotong University School of Medicine, Shanghai, China

**Keywords:** frozen-thawed embryo transfer, hormone replacement therapy, progesterone, live birth, birthweight

## Abstract

**Backgrounds:**

Previous studies suggested that singletons from frozen-thawed embryo transfer (FET) were associated with higher risk of large, post-date babies and adverse obstetrical outcomes compared to fresh transfer and natural pregnancy. No data available revealed whether the adverse perinatal outcomes were associated with aberrantly high progesterone level from different endometrium preparations in HRT-FET cycle. This study aimed to compare the impact of progesterone intramuscularly and vaginally regimens on neonatal outcomes in HRT-FET cycles.

**Methods:**

A total of 856 HRT-FET cycles from a fertility center from 2015 to 2018 were retrospectively analyzed. All patients had their first FET with two cleavage-staged embryos transferred. Endometrial preparation was performed with sequential administration of estrogen followed by progesterone intramuscularly 60 mg per day or vaginal gel Crinone 90 mg per day. Pregnancy outcomes including live birth rate, singleton birthweight, large for gestational age (LGA) rate, small for gestational age (SGA) rate, and preterm delivery rate were analyzed. Student’s t test, Mann-Whitney U-test, Chi square analysis, and multivariable logistic regression were used where appropriate. Differences were considered significant if p < 0.05.

**Results:**

No significant difference of live birth rate was found between different progesterone regimens (Adjusted OR 1.128, 95% CI 0.842, 1.511, p = 0.420). Neonatal outcomes like singleton birthweight (p = 0.744), preterm delivery rate (Adjusted OR 1.920, 95% CI 0.603, 6.11, p = 0.269), SGA (Adjusted OR 0.227, 95% CI 0.027, 1.934, p = 0.175), and LGA rate (Adjusted OR 0.862, 95% CI 0.425, 1.749, p=0.681) were not different between two progesterone regimens. Serum P level >41.82 pmol/L at 14 day post-FET was associated with higher live birth rate than serum P level ≤41.82 pmol/L in HRT-FET cycles when progesterone was intramuscularly delivered (Adjusted OR 1.690, 95% CI 1.002, 2.849, p = 0.049). But singleton birthweight, preterm delivery rate, SGA and LGA rate were not different between these two groups.

**Conclusions:**

Relatively higher serum progesterone level induced by intramuscular regimen did not change live birth rate or neonatal outcomes compared to vaginal regimen. Monitoring serum progesterone level and optimizing progesterone dose of intramuscular progesterone as needed in HRT-FET cycles has a role in improving live birth rate without impact on neonatal outcomes.

## Introduction

As cryopreservation has been an efficient and reliable laboratory procedure, freeze-all policy and elective embryo cryopreservation have been increasingly prevalent with a variety of clinic indications like preventing OHSS, implantation of PGT-A, fertility preservation, etc (1). There is a growing number of FET cycles applied due to the endometrial synchrony and relative steady endocrine environment compared to supraphysiologic estrogen milieu generated by controlled ovarian stimulation (COS) in fresh IVF cycles. However, we must recognize the adverse perinatal outcomes of FET, like higher risk of macrosomia, perinatal mortality, and pregnancy complications (2–5). Reports suggested that singletons from FET were associated with higher risk of large and post-date babies, placenta accrete, pre-eclampsia compared to fresh transfer and natural pregnancy (6–9). The reason for that varied. Some reports suggested embryo cryopreservation altered epigenetics regulation and induced abnormal placentation and fetal growth (7). Some studies advised that excessive estrogen priming set off the obstetrics complications (10, 11). Nevertheless, scarce literature has been focused on the supraphysiologic progesterone exposure to the deep placentation.

Progestin directly advanced vascular proliferation during placentation (12). A supraphysiologic progestin exposure in HRT could initiate excessively deep placentation which would make a difference in infant birthweight and obstetrical consequences (13). Previous studies explored the perinatal outcomes in endometrium preparation and indicated an inferior live birth rate with more obstetrics complications like hypertensive disorder, placenta accrete, post-term birth, and macrosomia in artificial cycle FET than nature cycle FET. They assumed a link between adverse obstetric outcomes and the lack of secretion of endogenous progesterone by functional corpus luteum (14–16). But there is no study investigating whether the adverse obstetrics outcome was related to different methods of endometrium preparation in HRT-FET cycles, especially aberrantly high circulating progesterone value induced by suboptimal dose and route of progesterone. The regimen and amount of progesterone applied in HRT-FET cycles varied a lot from different IVF centers, and the exogenous progesterone administered in HRT usually exceeds the endogenous progesterone of menstrual cycles by folds. There is little agreement on the ideal route and dose of administration. Optimizing it could be crucial to maximize both the clinical and neonatal successful rate. Thus our study aimed to explore whether circulating progesterone values induced by different progesterone regimens impact the live birth rate and neonatal outcomes.

## Methods

### Participants

The retrospective study was undertaken at the assisted reproduction medicine department in Shanghai General Hospital affiliated to Shanghai Jiao Tong University School of Medicine, including 856 women who had undergone their first FET from January 2015 to December 2018. Inclusion criteria were maternal age <48, undergoing two Day 2 or Day 3 cleavage-stage embryos transfer following HRT endometrium preparation. The patients with cryopreserved oocytes or donor oocytes and with prior attempts at conception *via* IVF and FET were excluded from the study. The final database included 856 women and 240 live birth singletons in the criteria. We kept patients re-examined in the first trimester and followed up by phone calls after the first trimester.

### IVF and Laboratory Protocols

Ovarian stimulation, oocyte retrieval, and IVF/ICSI procedures have been previously described (17). For IVF, oocytes were inseminated with human tubal fluid supplemented with 10% serum substitute supplement and with around 300,000 progressively motile spermatozoa. For ICSI, oocytes were placed in the fertilization medium immediately after microinjection. Fertilization was evaluated 18 h after insemination. Embryos were cultured in early cleavage medium before Day3 and in multiblast medium afterwards. All embryos were cultured in incubator at 37°C, 5% O_2_ and 6% CO_2_ concentration. Embryo development was evaluated on Days 2, 3, 5 and 6. Day 2 or Day 3 cleavage-stage embryos with at least two or six blastomeres respectively and with fragmentation <20% according to guidelines (18) were eligible for cryopreservation. The criteria for good-quality embryo were: four to six cells with less than 10% fragmentation for Day 2 embryos, seven to nine cells with less than 10% fragmentation for Day 3 embryos.

### Frozen Embryo Transfer Protocol

In a FET cycle, patients were administered estrogen and progesterone sequentially for endometrial preparation before FET. Patients started with estrogen administered orally (Estradiol Valerate or Estradiol Femoston) 6 mg per day with or without adding estrogen vaginally (Estradiol Femoston) 2 mg per day. Transvaginal ultrasonography, serum E2, LH, and progesterone level were measured at each visit weekly. Once the time of FET was determined, progesterone intramuscularly (60 mg per day) or vaginal gel Crinone (90 mg per day) was initiated daily. They both combined with dydrogesterone orally 30 mg per day and estradiol orally 6 mg per day. Intramuscular Progesterone or Vaginal Crinone was chosen according to patient preference after fully informing them with the advantages and side-effects of different routes such as vaginal itch and discharge or the subcutaneous swell experienced with intramuscular injection. Patients who were undertaken Day 2 or Day 3 cleavage-staged embryo transferred started the progesterone 2 or 3 days before FET, respectively. The vitrification and thawing procedure were previously presented (17). Embryo transfer was performed *via* the same flexible catheter under transabdominal ultrasound guidance. After FET, daily estrogen and progesterone administration continued until a negative pregnancy test was obtained at the 14th day after embryo transfer. If pregnancy was achieved, hormone administration continued until 12 weeks’ gestation.

### Outcome Measures and Definitions

In order to evaluate the impact of progesterone delivered regimens on clinical outcome, the primary outcome measure was live birth rate. Secondary outcome measures included clinical pregnancy rate, newborn birthweight, large for gestational age (LGA), small for gestational age (SGA), preterm delivery rate. Live birth was defined as a delivery of a viable infant after the 28th gestational weeks. Clinical pregnancy is a pregnancy confirmed by the confirmation of gestational sac or heartbeat. Gestational age was calculated from 14 days before the embryo transfer. Preterm birth was defined as delivery between 28 to 37 gestational weeks. SGA and LGA were defined as birthweight <10th and >90th percentile, respectively. Z score was administered to calculate birthweight adjusted for gestational age and newborn gender using the formula: Z score = (χ−μ) / σ, where χ is the birthweight of the infant, μ is the mean birthweight for the same sex and same gestational age in the reference group and σ is the standard deviation of the reference group. The reference is the Chinese singletons newborns (19).

### Statistical Analysis

Patients and singletons live birth demographic baseline, cycle characteristics, clinical and neonatal outcomes were compared using Student’s t-test, Mann-Whitney U test, chi-square, and Fisher’s exact tests, as appropriate. Whether binary live birth and clinical pregnancy were modified by the regimens of progesterone was assessed by multivariable logistic regression adjusting for major covariates as maternal age, BMI, the route of estrogen administered, whether duration of estradiol treatment >21 days, whether at least one good quality embryo was transferred. Multivariable logistic regression was applied to evaluate the regimens of progesterone impact on neonatal outcomes adjusting for the major covariates mentioned above plus newborn gender. Adjusted odds ratios (OR) and 95% confidence intervals (95% CI) were reported. All analyses were conducted with SPSS statistics. P value < 0.05 was considered statistically significant.

### Ethical Approval

Institutional review board and ethics committee of Shanghai General Hospital approval was obtained.

## Results

### Clinical Outcomes

This analysis included 856 women and 240 live birth singletons with the following outcomes: 44.04% clinical pregnancy rate, 35.63% live birth rate. There were 333 patients who were progesterone administered intramuscularly and 523 patients who were progesterone administered vaginally. Baseline demographics and characteristics were compared between patients with different progesterone regimens (**Table 1**). Among the 856 women, it did not reveal any significant differences for maternal age, BMI, whether there was at least one good quality embryo transferred, endometrium thickness at progesterone starting day, days of estrogen duration, E2, P, LH level at progesterone starting day between two groups. The proportion of patients with estradiol vaginally and orally delivered together in progesterone vaginally group was significantly higher than in progesterone intramuscularly group. Serum progesterone level at 14th day after embryo transfer was significantly higher in progesterone intramuscularly group than in progesterone vaginally group (40.5 pmol/L *versus* 14.95 pmol/L). No significant difference of live birth rate (Crude OR 1.181, 95% CI 0.895, 1.557, p = 0.282) and clinical pregnancy rate (Crude OR 1.170, 95% CI 0.879, 1.557, p = 0.239) was found between different progesterone regimens (**Table 2**). Controlling for maternal age, BMI, the route of estrogen administration, whether estradiol duration was longer than 21 days, whether there was at least one good quality embryo transferred, progesterone administered regimen did not modify the odds of achieving live birth (Adjusted OR 1.128, 95% CI 0.842, 1.511, p = 0.420) or clinical pregnancy (Adjusted OR 1.144, 95% CI 0.863, 1.518, p = 0.349) (**Table 2**). Maternal age and at least one good quality embryo transferred were the only independent factors that increased the live birth rate and clinical pregnancy rate.

**Table 1:** Baseline demographics and cycle characteristics according to different progesterone routes.

	Progesterone intramuscularly (N = 333)	Crinone vaginally (N = 523)	P
**Maternal age (y)**	30.46 ± 4.5	31.0 ± 4.5	0.051
**BMI**	21.47 ± 3.1	21.22 ± 3.0	0.26
**At least one good quality embryo**	294	451	0.383
**Endometrium thickness at P starting day (mm)**	9.0 (8.38, 9.63) (N = 320)	8.9 (8.4, 9.4) (N = 508)	0.355
**Days of estradiol administration**			
**>21 days**	24	36	0.856
**≤21 days**	209	487	
**Estrogen route**			
Orally and vaginally	126	229	0.009
Orally only	107	294	
**E2 level at P starting day (pmol/L)**	1374 (789.3, 3843.3) (N = 312)	1342.5 (822, 5291) (N = 479)	0.340
**P level at P starting day (pmol/L)**	1.1 (0.68, 1.7) (N = 311)	1.2 (0.65, 1.85) (N = 477)	0.663
**LH level at P starting day (pmol/L)**	8.8 (4.78, 14.11) (N = 311)	8.6 (4.44, 14.72) (N = 477)	0.561
**P level at 14th day after embryo transfer (pmol/L)**	40.5 (31.61, 57.57) (N = 262)	14.95 (8.29, 25.55) (N = 410)	<0.001

Data are presented as mean ± SD for continuous variables in formal distribution, median (first quartile, third quartile) for continuous variables in informal distribution. P values were assessed with the use of t tests or Wilcoxon rank sum tests or chi-square.

**Table 2:** Clinical outcomes according to different progesterone routes.

	Progesterone intramuscularly (N = 333)	Crinone vaginally (N = 523)	Crude OR(95% CI)	P	Adjusted OR(95% CI)	P
**Clinical pregnancy**	155 (46.55)	222 (42.45)	1.181 (0.895, 1.557)	0.239	1.144 (0.863, 1.518)^a^	0.349
**Live birth**	126 (37.84)	179 (34.23)	1.170 (0.879, 1.557)	0.282	1.128 (0.842, 1.511)^a^	0.420

a adjusted for maternal age, BMI, transfer with at least one good quality embryo, estrogen regimen, whether estrogen administration days >21. CI, confidence interval; OR, odds ratio.

### Neonatal Outcomes

To further explore the progesterone regimen impact on singleton birthweight and gestational age, a cohort of 240 live birth singletons from 856 patients was further investigated. Neonatal outcomes stratified by the regimens of progesterone administered were presented in **Table 3**. Newborn gender, gestational age, mean birthweight, Z-scores, preterm delivery rate, SGA and LGA rate were not different across two groups (**Table 3**). In multivariate analyses (**Table 3**), the risk of preterm delivery (Adjusted OR 1.920, 95% CI 0.603, 6.11, p = 0.269), the risk of LGA (Adjusted OR 0.862, 95% CI 0.425, 1.749, p = 0.681), and SGA (Adjusted OR 0.227, 95% CI 0.027, 1.934, p = 0.175) were not significantly different between two groups after adjusting for maternal age, BMI, estradiol route, whether estrogen administration lasted more than 21 days, whether at least one good quality embryo was transferred, and newborn gender.

**Table 3:** Perinatal outcomes of live birth singletons according to different progesterone routes.

All singletons	Progesterone intramuscularly (N = 95)			Crinone vaginally (N = 145)		P
**Newborn gender**						
**Female**	45			71		0.809
**Male**	50			74		
**Gestational age**						
**32-36**	7			7		0.411
**≥37**	88			138		
**Birthweight**	3349.19 ± 487.2			3365.58 ± 469.5		0.744
**Z score**	0.357 ± 1.047			0.345 ± 1.023		0.928
	**Progesterone intramuscularly (N = 95)**	**Crinone vaginally (N = 145)**	**Crude OR** **(95% CI)**	**P**	**Adjusted OR** **(95% CI)**	**P**
**Preterm delivery**	7	7	1.568 (0.532, 4.623)	0.411	1.920 (0.603, 6.110)^a^	0.269
**SGA**	1	6	0.246 (0.029, 2.080)	0.319	0.227 (0.027, 1.934)^a^	0.175
**LGA**	14	27	0.819 (0.410, 1.637)	0.572	0.862 (0.425, 1.749)^a^	0.681

Data are presented as mean ± SD for continuous variables in formal distribution. P values were assessed with the use of t tests or Mann-Whitney U tests or chi-square (Fisher’s exact tests as appropriate).

a adjusted for maternal age, BMI, transfer with at least one good quality embryo, estrogen regimen, whether estrogen administration days >21, newborn gender. SGA, small for gestational age; LGA, large for gestational age.

### Subgroup Analysis

In order to investigate the circulating serum progesterone impact on singleton gestational weeks and birthweight, a cohort of 262 patients with progesterone intramuscularly administered and 77 live birth singletons was further investigated. We did not analysis vaginal progesterone cohort because systemic serum progesterone value was not the reflection of progesterone Crinone vaginally absorbed (20). From the cohort, clinical and neonatal outcomes of patients with serum progesterone level >41.82 pmol/L and ≤41.82 pmol/L at the 14th day after embryo transfer were compared in **Table 4**. The median value of serum progesterone level was 41.82 pmol/L at the 14th day after embryo transfer for patients with progesterone intramuscularly administered. Patients with serum P level >41.82 pmol/L demonstrated higher clinical pregnancy rate (Adjusted OR 1.670, 95% CI 1.005, 2.774, p = 0.048) and higher live birth rate (Adjusted OR 1.690, 95% CI 1.002, 2.849, p = 0.049) than patients with serum P level ≤41.82pmolL both in univariate analysis and multivariate analysis adjusting for maternal age, BMI, whether at least one good quality embryo was transferred, estrogen regimen, whether estrogen administration days >21. While birthweight, Z-score, preterm delivery rate, LGA and SGA rate were not different between these two groups. Multivariate analysis was not performed for preterm delivery rate, LGA and SGA rate because the sample size in this category was too small.

**Table 4:** Clinical and perinatal outcomes between P level >41.82 pmol/L and P level ≤41.82 pmol/L groups when progesterone intramuscularly was used in HRT-FET cycles.

	P level >41.82 pmol/L (N = 131)	P level ≤41.82 pmol/L (N = 131)	Crude OR(95% CI)		P	Adjusted OR(95% CI)	P
**Clinical Pregnancy**	72 (54.9)	55 (41.98)	1.686 (1.034, 2.940)		0.036	1.670 (1.005, 2.774)^a^	0.048
**Live Birth**	59 (45.04)	43 (32.82)	1.677 (1.016, 2.769)		0.043	1.690 (1.002, 2.849)^a^	0.049
**Singletons Neonatal outcome**	**P level >41.82 pmol/L (N = 42)**	**P level ≤41.82 pmol/L (N = 35)**	**Crude OR** **(95% CI)**			**P**	
**Birthweight**	3331.79 ± 421.19	3354.03 ± 643.51				0.861	
**Z score**	0.2778 ± 0.9658	0.6248 ± 1.1147				0.153	
**Preterm delivery**	2	5	0.391 (0.074, 2.051)			0.444	
**LGA**	5	8	0.610 (0.194, 1.917)			0.393	
**SGA**	1	0	/			/	

a adjusted for maternal age, BMI, transfer with at least one good quality embryo, estrogen regimen, whether estrogen administration days >21. SGA, small for gestational age; LGA, large for gestational age.

## Discussion

### Main Findings

From our study, we found no difference of live birth rate, clinical pregnancy rate, singleton birthweight, preterm delivery rate, LGA and SGA rate between progesterone vaginally and intramuscularly administrations in HRT-FET cycles. Relatively higher serum progesterone level induced by intramuscular regimen did not increase newborn birthweight or prolong gestational weeks compared to vaginal regimen. As for intramuscular progesterone supplementation, serum progesterone concentration higher than 41.82 pmol/L at day 14 post-FET was associated with improved live birth rate and comparable neonatal outcomes compared to P level ≤41.82 pmol/L. Monitoring serum progesterone level and optimizing progesterone dose as needed in intramuscular progesterone HRT-FET cycles has a role in improving clinical outcomes without impact on perinatal outcomes.

### Interpretation of Data

Previous studies indicated FET resulted in increased risk of pregnancy-induced hypertension, LGA and post-date newborns compared to fresh IVF-ET cycles (2, 6–8). The difference of gestational weeks and birthweight between FET singletons and fresh IVF cycles singletons led people to speculate the association of the quality of placentation and hormone characteristics from different cycles. People presumed the reasons behind this phenomenon involved the supraphysiologic estrogen milieu in ovarian stimulated cycles (10, 11), embryo cryopreservation technique *per se* induced epigenetics alteration (7), and the lack of functional corpus luteum in HRT-FET cycles (14–16). However no data available explored whether newborn gestational age, birthweight, and the placental-related obstetric complications were associated with the specific protocols of endometrium preparation in FET cycles, especially progesterone replacement regimens. In HRT-FET cycles, once the adequate proliferation of the endometrium is achieved, progesterone daily starts before scheduled embryo transfer, which elicits decidualization of estrogen-primed endometrial stromal cells and develops endometrial receptivity. Progestin assists with extravillous trophoblast (EVT) invasion and endometrium vascular remodeling, which are important for pregnancy, because defects in extravillous trophoblast invasion could generate first trimester decidual hemorrhage and induce later adverse outcome like pre-eclampsia and post-date newborn (21). Progesterone acts on endometrium inhibiting uterus contractility and creates uterine quiescence (22). There is no functioning corpora luteal and endogenous progesterone production in HRT. The progesterone replacement applied in FET cycles usually created a relatively high P milieu. Several studies reported the negative impact of very high progesterone level on endometrium maturation and implantation in non-human studies (23, 24). Aberrantly higher levels of progesterone in early pregnancy can result in over-invasion of the extravillous trophoblast invasion by affecting the functions of syncytial trophoblast and decidual cells, in this way potentiating the later superficial placentation which influence newborn birthweight and gestational weeks (12, 21, 25).

The ideal administration route, duration, and the dosage of progesterone in HRT-FET cycles have not been well defined. Vaginal Crinone and intramuscular progesterone are two preferred luteal support regimens. Vaginally delivered progesterone reached the uterus directly and induced higher progesterone concentration in the uterine endometrium with relatively lower circulating P level compared to intramuscular regimen (26). Report has suggested micronized vaginal P supplement helped decrease uterine contraction frequency and lowered the risk of displacement of embryo in fresh IVF-ET cycles (27). While vaginal progesterone administration for luteal support has provided strong evidence of similar pregnancy and birth outcomes compared to intramuscular P in fresh IVF cycles (28–30). However in FET cycles, the successful rate is in favor of progesterone intramuscular administration compared to vaginal and oral administration (1, 31, 32). It is noteworthy that comparison between different routes of progesterone administered is beneficial in clarifying not only the most effective but also the relative safer one. Here our research focused on whether large-spanned circulation progesterone values elicited by different luteal support regimens made a difference in both successful clinical outcome and neonatal outcomes including newborn gestational weeks and birthweight.

From our study, we showed that progesterone vaginally administered resulted in comparable live birth rate with intramuscular regimen as luteal support in HRT-FET cycles, in line with similar results found in fresh IVF cycles (33–35). We further analyzed neonatal outcomes stratified by different progesterone regimens and found no significant difference of infant birthweight, preterm delivery rate, SGA and LGA rate between two groups. Although the higher risk of LGA and post-date newborns was found in FET singletons compared to fresh ET cycles (2, 6–8), and study suggested the aberrantly higher levels of progesterone resulted in over-invasion of the EVT which intervened the perinatal outcome (12), relatively higher peripheral serum progesterone level by intramuscularly delivered progesterone did not make higher birthweight and longer gestational weeks compared to vaginal regimen in our cohort. The reason may be speculated that the placenta formation and angiogenesis could be influenced by not only circulating progesterone level, but also uterine local progesterone level. Vaginally delivered progesterone gel might create the high concentration of progesterone at the maternal-fetal interface but it is hard to detect by blood drawing. In our cohort patients received dydrogesterone orally at the same time in case of vaginal malabsorption, though dydrogesterone orally taken did not contribute to the serum progesterone value either. And it shouldn’t be neglected that vaginal progesterone uptake distribution and metabolism vary tremendously between patients. Additionally, it could be the lack of corpus luteum and the absence of vasoactive hormones like Relaxin released from corpus luteum in HRT-FET cycles that play the dominant role in the adverse perinatal outcomes (16, 36). So the different extent of high progesterone level induced by different progestin replacement did not change the newborn birthweight and gestational weeks in our analysis. Our study compared specific protocols of endometrial preparation used for artificial FET cycles and added some evidence supporting that large-spanned circulating progesterone levels induced by different progesterone routes didn’t change the neonatal outcomes like singleton birthweight and gestational age.

Some studies showed low serum progesterone on the day of embryo transfer is associated with inferior clinical pregnancy both in artificial FET and fresh IVF-ET cycles (37–39). In our study we analyzed the circulating progesterone concentration of 14th day after embryo transfer in HRT-FET cycles, which is also hCG test day. At this time almost no endogenous progesterone from placenta is present. Only little progesterone from trophoblasts contributes to serum progesterone concentration in the peri-implantation period. Our results showed when intramuscularly delivered progesterone was applied as luteal support, higher serum progesterone level at 14th day after embryo transfer got higher successful rate than the lower P level counterparts in HRT-FET cycles. Thus clinicians could optimize intramuscular progesterone supplementation according to the P monitoring. Our findings confirmed previous data that luteal progesterone level outside the range limits reduced clinical pregnancy rate (40–42). But we further explored newborn birthweight and gestational age in this intramuscular progesterone cohort and revealed that systemic P level higher than 41.82 pmol/L did not increase birthweight or prolong gestational weeks, but it increased the live birth rate. Thus it is advised to monitor systemic P concentration and provide higher progesterone dose as needed in intramuscular progesterone patients to optimize the live birth rate without impact on neonatal outcomes. Increasing the dose of intramuscular progesterone when circulating P value is lower than 41.82 pmol/L was presumed to be a superiority. We did not analyze the circulating progesterone value impact in the vaginal progesterone cohort because circulating progesterone concentration does not serve as a surrogate marker for the amount of vaginal progesterone absorbed (20).

### Strengths and Limitations

The present study has following strengths. The analysis was performed in one single IVF center which guaranteed the same laboratory procedures and sonographers. The analysis only included patients with their first FET cycles to assure the relative good quality embryo transferred and exclude the recurrent embryo failure cases. We excluded blastocyst transfer to alleviate prolonged *in vitro* embryo culture impact on neonatal outcomes (43, 44). To control for infant gender and gestational age bias, z score was calculated across two groups. There were limitations in this study. It is a retrospective analysis and selection bias was possible. Although we accounted for some associated factors in multivariate analysis, unknown factors might have affected the results. Large randomized prospective study is needed. We couldn’t follow up the patient about obstetrics details, which impeded us to assess perinatal outcomes like gestational hypertension, pre-eclampsia, placenta accrete and previa. The obstetrics details would allow us a better understanding of high serum progesterone level’s impact on placental formation in pregnancy. Additionally, in order to analyze whether obstetrics inferiority in HRT-FET was associated with a lack of secretions of endogenous progesterone by functional corpus luteum or a suboptimal dose or route of exogenous progesterone administration, it is better to address this issue by comparing perinatal outcomes between different progesterone regimen protocols in artificial cycles as well as natural cycles.

## Conclusion

In conclusion, this is the first study to demonstrate the association of progesterone regimen protocols and neonatal outcomes. Relatively higher serum progesterone level induced by intramuscular regimen did not change live birth rate, or increase newborn birthweight or prolong gestational weeks compared to vaginal regimen. During intramuscular progesterone supplementation of HRT-FET cycles, circulating progesterone concentration higher than 41.82 pmol/L at day 14 post-FET was associated with improved live birth rate. Monitoring serum progesterone level and optimizing progesterone dose as needed in intramuscular progesterone HRT-FET cycles has a role in improving clinical outcomes without impact on neonatal outcomes.

## Data Availability Statement

The datasets generated for this study are available on request to the corresponding author.

## Ethics Statement

The studies involving human participants were reviewed and approved by the institutional review board and ethics committee of Shanghai General Hospital. The ethics committee waived the requirement of written informed consent for participation.

## Author Contributions

YL and YW were involved in study concept and design. YL collected and analyzed the data. YL drafted the article. YW revised it for important intellectual content. All authors contributed to the article and approved the submitted version.

## Funding

This study was supported by a grant from the National Natural Science Foundation of China (No. 82002738). Money was to appreciate the hard work of all authors.

## Conflict of Interest

The authors declare that the research was conducted in the absence of any commercial or financial relationships that could be construed as a potential conflict of interest.

## Abbreviations

HRT, hormone replacement therapy; FET, frozen-thawed embryo transfer; COS, controlled ovarian stimulation; IVF, *in vitro* fertilization; ICSI, intracytoplasmic sperm injection; LGA, large for gestational age; SGA, small for gestational age; EVT, extravillous trophoblast; 95% CI, 95% confidence intervals; OR, odds ratios; BMI, body mass index.
